# Large Foetus

**Published:** 1914-05

**Authors:** 


**Large Foetus.** Dr. Ella F. Gatchell reports, Calif. State Jour. of Med., the birth by breech presentation of a boy weighing 18 pounds, 23 inches tall, chest circumference 17 inches, head 15. The father weighed 160, the mother, herself delivered by Caesarian section, 130.

